# The Life-Cycle of the Guinea-Worm

**Published:** 1910-03-05

**Authors:** 


### Tropical Medicine.

### THE LIFE-CYCLE OF THE GUINEA-WORM.

The following conditions are necessary for the propagation of the guinea-worm: The young must be discharged directly into fresh water soon after the parent worm has succeeded in creating a break in the overlying skin of the infected patient and before the wound has become markedly septic. The embryos must find a cyclops within a few days. They must, moreover, succeed in entering its body cavity. Five weeks later they will have developed into mature larvæ. These must thereafter be taken into the human stomach, and having been set free from their host by the gastric juice, reach the connective tissues by penetrating the gut wall. The life-cycle of the parasite will necessarily be broken: (1) By the death of the embryos, either from sepsis while still within the parent worm, or, if after their discharge, by saltish water or by drying. (2) If no cyclops is present in the water, or if the infected cyclops dies or is not taken into the human stomach. (3) If the larvæ, ingested by the final host, are immature or fail to escape from the chitinous sheath of the cyclops. Though they do find their final habitat, the cycle will still be incomplete if (4) there are not both males and females among the matured adults and if in their wandering the females are not impregnated.

It will at once be seen from the above summary that the isolation of infected men from healthy cyclops and of infected cyclops from man must be the object of any organised effort to stamp out dracontiasis.

